# The Mediterranean Diet as a Source of Natural Compounds: Does It Represent a Protective Choice against Cancer?

**DOI:** 10.3390/ph14090920

**Published:** 2021-09-11

**Authors:** Giuseppina Augimeri, Daniela Bonofiglio

**Affiliations:** 1 Department of Pharmacy, Health and Nutritional Sciences, University of Calabria, 87036 Arcavacata di Rende, CS, Italy; giuseppina.augimeri@unical.it; 2 Centro Sanitario, University of Calabria, 87036 Arcavacata di Rende, CS, Italy

**Keywords:** Mediterranean Diet, healthy eating, cancer prevention, cancer incidence

## Abstract

The Mediterranean diet (MD), characterized by a high intake of fruits, vegetables, legumes, nuts and grains, a moderate intake of red wine and a reduced consumption of meat, has been considered one of the healthiest dietary patterns worldwide. Growing evidence suggests an inverse relationship between high adherence to the MD and cancer, as well as other chronic degenerative diseases. The beneficial effects elicited by the MD pattern on cancer are due to the high contents of bioactive compounds contained in many foods of MD, which protect cells by oxidative and inflammatory processes and inhibit carcinogenesis by targeting the various hallmarks of cancer with different mechanisms of action. Although over the past decades numerous dietary and phytochemical compounds from Mediterranean food that have anticancer potential have been identified, a clear association between the MD eating pattern and cancer needs to be established. While we wait for answers to this question from well-conducted research, the empowering of the MD as a protective choice against cancer should represent the priority for public health policies.

## 1. Introduction

Cancer is considered one of the most serious issues affecting humans, being the second leading cause of mortality after cardiovascular diseases worldwide. Overall, the burden of cancer incidence and mortality is rapidly growing globally and the International Agency for Research on Cancer and the World Health Organization predicts that by 2040 there will be approximately 30 million cases of cancer with an annual mortality rate of 16.3 million individuals [1]. The development of cancer has been associated with several factors, such as genetic, hormonal, environmental and nutritional factors, which can act directly or indirectly stimulating the expression of the malignant phenotype that regulates its progression. Over the past few decades, a surge of cancer cases has been largely related to human activities ranging from unhealthy individual lifestyles to a negative global environmental impact, all of which can be modified. Approximately 30–35% of cancer cases are associated with dietary factors able to act in the prevention of cancer development and progression, by making cancer a preventable disease [2,3]. Evidence from clinical trial outcomes and epidemiological observations has all provided clues about the biology of cancer prevention and for the relationship between diet and cancer [4,5,6]. Dietary patterns characterized by regular intake of fruit and vegetables, which are rich in minerals, vitamins and other essential substances, collectively called phytochemicals, play a protective role in cancer onset reducing the risk of most of the neoplasms [6,7]. A high intake of whole grain fibers, as a source of phytoestrogens, such as lignans and isoflavones, may decrease the incidence of different types of cancer, mainly of the digestive system [8,9]. On the contrary, intake of animal fat or red meat, often cooked at high temperatures, may increase cancer incidence, especially for colorectal and prostate cancer [10,11]. Particularly, gastric cancer, only case-control studies showed positive effects between red meat consumption and cancer risk, whereas null results were observed in cohort studies [12].

Combining this evidence, the diet quality defined by dietary pattern represents a reliable index for healthy nutrition. Among the dietary patterns, the Mediterranean Diet (MD) is recognized as one of the healthiest eating models currently under consideration in the field of cancer prevention since MD is a combination of foods that contain a wide spectrum of bioactive dietary components or nutrients with antineoplastic properties. Herein, we briefly discuss the evidence available on the potential antitumoral effects of the dietary compounds from the MD, highlighting the importance to adopt this healthy eating model as a protective choice against cancer.

## 2. Natural Compounds from the Mediterranean Diet: Molecular Mechanisms in Hallmarks of Cancer

**Figure 1:**
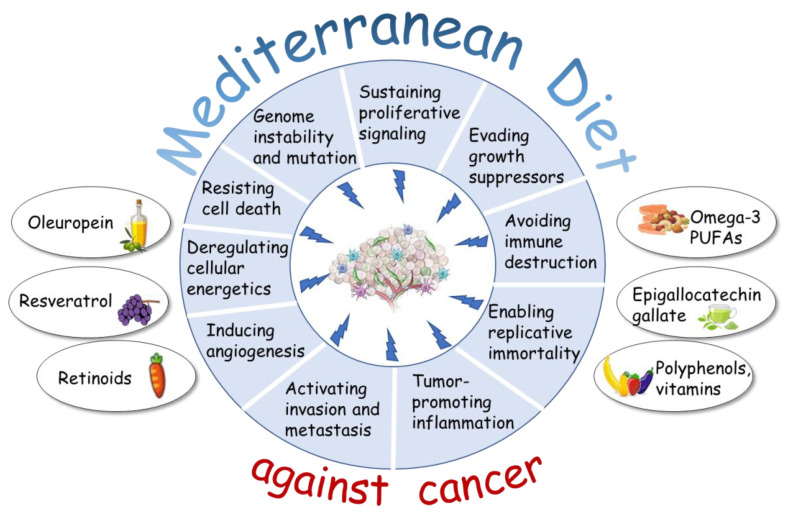
Schematic representation of the molecular mechanisms by which Mediterranean Diet (MD) pattern may impact on hallmarks of cancer. Foods of the MD, including olive oil, red wine, fruits, legumes and vegetables, contains bioactive molecules such as oleuropein, resveratrol, retinoids, vitamins, epigallocatechin-gallate and omega-3 polyunsaturated fatty acids (PUFAs), that exert anti-tumoral activities by targeting the various hallmarks of cancer.

The term Mediterranean Diet pattern [13] was firstly introduced in 1975 by Ancel Keys based on the traditional eating habits of people from countries that surround the Mediterranean Sea. In the following decades, a plethora of studies examined the health effects of this diet model, which is characterized by a high intake of vegetables, legumes, fresh or dried fruits, low fat dairy products, a moderate intake of fish and poultry, along with a low intake of red meat, processed foods and sweets, all accompanied by daily using of extra virgin olive oil and herbs and a moderate consumption of wine. It is recommended drinking teas and infusions of aromatic herbs, since they are enriched of phenolic compounds with healthy benefits [14]. In 2010, since MD “constitutes a set of skills, knowledge, practices and traditions ranging from the landscape to the table, including the crops, harvesting, fishing, conservation, processing, preparation and, particularly, consumption of food”, the United Nations Educational, Scientific and Cultural Organization (UNESCO) recognized this dietary pattern, derived from a complex interaction of diet with culture, as “intangible cultural heritage of humanity” [15]. The beneficial effects of the MD in cancer prevention have been associated to the presence of bioactive compounds in many foods of this dietary pattern that are commonly referred as nutraceuticals. Plant-based foods, including fruits, vegetables, cereals, nuts and seeds, legumes and vegetable oils, are the main source of antioxidant nutrients, such as vitamins A, C, E, carotenoids, lycopene, the trace mineral selenium and many other minerals which can protect the cells against damage, reducing the malignant process and even reversing it [16]. Additionally, plant-based foods contain numerous phytochemicals, such as polyphenols, isoflavones, phenolic acids, terpenes, retinoids, while in green tea and in red wine are present components known as epigallocatechin gallate and resveratrol, respectively, which have anticarcinogenic properties [17,18]. Regarding the extra virgin olive oil, it has been hypothesized that its chemo-preventive potential is due to the antioxidant and antiproliferative activities of the polyphenolic content rich in hydroxytyrosol, tyrosol and secoiridoid derivatives, such as oleuropein and oleocanthal [19]. Interestingly, plant-based phenolic compounds also modulate insulin actions improving insulin resistance which is a risk factor for several types of cancer [20].The mechanisms by which several bioactive food components of the MD, such as polyphenols, carotenoids, catechins, polyunsaturated fatty acids (PUFAs), may affect many cellular signaling pathways involved in tumorigenesis emerged from numerous in vitro and in vivo studies which provide evidence for their ability to target various hallmarks of cancer with different mechanisms of action [17,18,21,22,23]. Specifically, natural compounds, including resveratrol, retinoids, epigallocatechin-gallate and omega-3 PUFAs, exert their anticancer activity by inducing cell cycle arrest in G0/G1, G2/M and S phases by activating p53 and p21 signaling pathways as well as the proapoptotic proteins Bcl-2-associated X protein (Bax) and Bcl-2 homologous antagonist killer (Bak) by downregulating the antiapoptotic protein B-cell lymphoma 2 (Bcl-2). In addition, they can decrease phosphorylation of the epidermal growth factor receptor (EGFR) can inhibit enzymes, such as cyclooxygenase-2 (COX-2) and lipo-oxygenases, as well as inhibit signal transduction enzymes, such protein kinase C (PKC), phosphatidyl-Inositol 3-Kinase (PI3K) and its downstream AKT signaling pathways. Moreover, some nutraceuticals, such as eicosapentaenoic acid (EPA), docosaxaenoic acid (DHA) and resveratrol mainly found in fish and grapes, respectively, also may reduce the expression of proteins associated with inflammatory process (NFκB, TNF-α IL-6, IL-1β) as well as blood vessel formation (VEGF, HIF) [24]. Thus, these molecules, inhibiting signaling pathways involved in proliferation, inflammation, invasion, angiogenesis, metastasis and activating death processes such as autophagy and apoptosis, show the potential ability to target important hallmarks of cancer cells. However, current evidence regarding the impact of only plant-based dietary patterns on cancer-related outcomes needs to be further investigated [25]. Importantly, dietary components of the MD also include long-chain omega-3 PUFAs from fresh fish, nuts and seed oils, which exert anti-inflammatory effects and anti-neoplastic activities by inducing autophagy and apoptotic cell death in human cancer cells, including breast cancer (reviewed in [24]). The proposed main routes of the pleiotropic and multifaceted actions of omega-3 PUFAs and their derivatives are related to changes in the distribution and function of key survival and death signals, the increased levels of intracellular oxidative stress, the binding to nuclear receptors, such as the tumor suppressor Peroxisome Proliferators-Activated Receptors (PPARs) leading to cancer cell death [26,27]. Overall, the MD pattern may be considered as a nutritional pool which includes bioactive components present in the typical foods and capable of exerting a protective role against cancer. A large number of cancer research studies focused on the potential effects of natural compounds from the MD in targeting several hallmarks of cancer cells, which include sustaining proliferative signaling, evading growth suppressors, avoiding immune destruction, enabling replicative immortality, increased tumor-promoting inflammation, activating invasion and metastasis, inducing angiogenesis, deregulating cellular energetics, resisting cell death, increased genomic instability and mutations (Figure 1).

## 3. Mediterranean Diet: A Healthy Lifestyle to Be Empowered

Although the potential benefits of single dietary bioactive compounds of the MD on cancer has been widely described, we want to underscore that the association between the MD eating pattern and cancer needs to be further explored. Indeed, the beneficial effects of the MD on health are not related to individual foods or components of the MD, but likely depend on the combination of foods and the interaction between its components. This concept is called food synergy which is sustained by dietary variety and by the choice of nutrient-rich foods to prevent chronic diseases [28,29]. To evaluate the whole diet pattern and establish the compliance with the MD, several tools have been developed over the last decades. To date, more than 20 indexes, based on questionnaires designed to investigate the frequency of pattern-consistent and -inconsistent food consumption, are available to quantify the adherence to the MD and its benefits regarding health [30]. Using these tools, it has been demonstrated that greater adherence to the MD is associated with a significant reduction in overall mortality and in morbidity from a wide spectrum of chronic diseases, such as obesity, type 2 diabetes, metabolic syndrome, neurogenerative and cardiovascular diseases and cancer [31,32,33,34,35]. With regards cancer outcomes, the highest score to the MD was associated with lower mortality risk for colorectal, gastric, liver, head and neck and breast cancer [36]. More recently, updated evidence on this inverse association had suggested the reduction of cancer risk mortality in the general population and the decrease in overall death rates among cancer survivors who adhere to the MD [37]. However, some studies showed difficulty to identify the association between MD and cancer [35,38,39]. The lack of clear evidence on the relationship between MD and reduced cancer risk could be due to several factors, including the low number of studies analyzing this association, the low sample size, the use of a wide variety of MD scores, the different study designs as well as the lack of very long term well designed cohort studies required for effective/stronger relative risk (RR) and hazard risk (HR) estimations [31]. In addition, other factors, such as the geographical localization, the growing and storage conditions of the Mediterranean foods, influence the content of bioactive compounds, making difficult the possibility to associate the potential effects of the dietary pattern with cancer onset [40]. Moreover, since MD is not just a diet, but a healthy lifestyle, in addition to the dietary intake, other parameters, such as physical activity, should be considered to evaluate the adherence to the MD and its impact on cancer risk. Therefore, additional clinical trials, systematic reviews and meta-analyses are needed to provide conclusive evidence for a beneficial effect of high adherence to the MD with respect to protecting role against cancer. While we wait for answers to this question from well-conducted research, the adherence to the MD as a protective choice against cancer should be strengthened. Indeed, over the last years, the adherence to the MD pattern is progressively decreasing because of several factors, including the dissemination of the Western dietary models and the food globalization [41]. Moreover, the enhanced cost of many foods of the MD has contributed to driving people to buy less expensive foods that have typically more calorie intake and lower nutritional quality. In order to ameliorate dietary quality, an important action is represented by “the Farm to Fork” strategy that addresses the challenges of sustainable food systems which is linked with healthy population and healthy planet. In addition, the lack of information about the quality of the foods seems to be a key factor for the decreased adherence to the MD. To reverse this trend and enhance the adherence to the MD, nutrition education and culinary skills should be provided especially to the younger generations. Recently, we demonstrated that the nutrition education program related to knowledge of food sources of macro- and micronutrients included in the healthy MD pattern improved the MD adherence in adolescents [42]. Interestingly, optimal adherence to the MD decreased inflammatory status as well as reduced the oxidative stress in adolescents, highlighting the positive impact of healthy eating as a strategy for the prevention of chronic-degenerative diseases over the entire lifespan [43].

## 4. Conclusions

Despite the clear evidence on the anticancer properties of natural compounds from the MD, the association between nutrition and cancer risk by clinical endpoints should be further explored in randomized trials or observational studies. However, while research will be carried out, the empowerment of the MD should represent the priority for public health policies. Specifically, it becomes mandatory to increase population awareness towards the promotion of the MD as a global nutritionally balanced and healthy dietary pattern and as a protective choice against cancer.

## Data Availability

Data sharing not applicable.
